# Electronic Stethoscope Auscultation and Echocardiography in ARDS: Correlation and Prognostic Value for Mortality and ICU Length of Stay: A Prospective Observational Study

**DOI:** 10.3390/medicina62030470

**Published:** 2026-03-01

**Authors:** Ioannis Alevroudis, Serafeim-Chrysovalantis Kotoulas, Christina Mouratidou, Aliki Karkala, Anastasia Michailidou, Myrto Tzimou, Spyridon Synodinos-Kamilos, Chrysavgi Giannaki, Christos Karachristos, Athina Lavrentieva, Nicos Maglaveras, Evangelos Kaimakamis

**Affiliations:** 1 Adult ICU, General Hospital of Thessaloniki Ippokrateio, School of Medicine, Aristotle University of Thessaloniki, 62500 Serres, Greece; akiskotoulas@hotmail.com (S.-C.K.); chris1mourat@gmail.com (C.M.); 2 3rd Cardiology Department of Aristotle University of Thessaloniki, General Hospital of Thessaloniki Ippokrateio, 54642 Thessaloniki, Greece; 3 Department of Pulmonology, Leuven University Center for Sleep and Wake Disorders (LUCS), University Hospitals Leuven Campus Gasthuisberg, Herestraat 49, 3000 Leuven, Belgium; aliki.karkala@uzleuven.be; 4 2nd Propaideutic Internal Medicine Departement, General Hospital of Thessaloniki Ippokrateio, 54642 Thessaloniki, Greece; manastasia96@gmail.com; 5 1st Intensive Care Unit, “G. Papanikolaou” General Hospital, 57010 Thessaloniki, Greece; tzimzapatista@gmail.com (M.T.); soullaroi@gmail.com (S.S.-K.); giannakichrysavgi@hotmail.com (C.G.); christosk2004@yahoo.gr (C.K.); alavrenti@gmail.com (A.L.); vakaimak@yahoo.gr (E.K.); 6 2nd Department of Obstetrics and Gynecology, The Medical School, Aristotle University of Thessaloniki, 62500 Serres, Greece; nicmag@auth.gr

**Keywords:** acute respiratory distress syndrome, cardiac auscultation, echocardiography, electronic stethoscope, right ventricular systolic pressure, SOFA score, intensive care unit, mortality prediction

## Abstract

*Background and Objectives*: Acute respiratory distress syndrome (ARDS) carries high mortality, with cardiovascular complications frequently contributing to adverse outcomes. This study investigated the relationship between cardiac auscultation using electronic stethoscopy and echocardiographic findings and evaluated their prognostic significance in mechanically ventilated ARDS patients. *Materials and Methods*: This prospective observational study enrolled 173 consecutive adults with ARDS requiring mechanical ventilation (June 2020–June 2021). Cardiac auscultation was performed using an electronic stethoscope at four standard valvular positions. Bedside echocardiography assessed ventricular function, valvular regurgitation, right ventricular systolic pressure (RVSP), and inferior vena cava dimensions. Primary outcomes were ICU and 90-day mortality; the secondary outcome was ICU length of stay. *Results*: ICU mortality was 42.2% and 90-day mortality 46.8%. Auscultation findings correlated significantly with echocardiographic parameters: aortic stenosis murmur with an elevated aortic valve velocity (*p* = 0.009), and mitral/tricuspid regurgitation murmurs with corresponding color Doppler findings (*p* < 0.001). In multivariate analysis, the mean daily SOFA score (OR 2.39, 95% CI 1.57–3.64, *p* < 0.001) and RVSP (OR 1.07, 95% CI 1.02–1.11, *p* = 0.006) independently predicted ICU mortality. For 90-day mortality, the APACHE II score (OR 1.25, *p* = 0.006), mean daily SOFA score (OR 1.54, *p* = 0.039), RVSP (OR 1.07, *p* = 0.020), and mitral regurgitation severity (OR 2.98, *p* = 0.031) were independent predictors. ICU length of stay was predicted by the mean daily SOFA score (r = 0.35, *p* < 0.001) and tricuspid regurgitation severity (r = 0.25, *p* = 0.012). *Conclusions*: Electronic stethoscope auscultation correlates with the echocardiographic findings in ARDS patients. The RVSP and SOFA scores independently predict mortality, while valvular regurgitation severity provides additional prognostic information for long-term survival and ICU resource utilization.

## 1. Introduction

Acute respiratory distress syndrome (ARDS) remains a leading cause of morbidity and mortality in the intensive care unit (ICU), accounting for approximately 10% of all ICU admissions worldwide and 23% of patients requiring mechanical ventilation [1]. Despite advances in lung-protective ventilation strategies, prone positioning, and restrictive fluid management, mortality rates remain unacceptably high, ranging from 35% to 46% depending on disease severity [1,2]. The pathophysiology of ARDS extends beyond pulmonary dysfunction, with cardiovascular complications playing a critical role in determining patient outcomes. Hypoxic pulmonary vasoconstriction, inflammatory mediator release, and the hemodynamic effects of positive pressure ventilation frequently lead to right ventricular dysfunction and pulmonary hypertension, which may independently contribute to mortality [3,4].

Right ventricular injury, including dysfunction, failure, and acute cor pulmonale, is present in approximately 21% of patients with ARDS and has been associated with increased mortality [5,6]. A recent systematic review and meta-analysis demonstrated that the presence of right ventricular dysfunction or pulmonary vascular dysfunction in patients with ARDS was associated with a 45–68% increase in mortality (OR 1.45–1.68, 95% CI 1.13–2.32) [5,7]. Severe acute cor pulmonale, characterized by right ventricular dilatation with septal dyskinesia, is particularly associated with excess mortality and circulatory failure [4]. These findings underscore the importance of comprehensive cardiovascular assessment in critically ill patients with ARDS, as right heart failure represents a potentially modifiable risk factor.

Cardiovascular assessment in critically ill patients has evolved considerably over the past decades. Bedside echocardiography has become an essential tool in the ICU, enabling real-time evaluation of ventricular function, valvular abnormalities, and hemodynamic status [8]. Point-of-care ultrasound is now recommended by international guidelines from the Society of Critical Care Medicine and the American Society of Echocardiography as a standard component of critical care assessment [9,10]. Echocardiographic parameters such as right ventricular systolic pressure (RVSP), ejection fraction, and the severity of valvular regurgitation provide valuable diagnostic and prognostic information. Studies in heart failure populations have demonstrated that elevated RVSP (≥40 mmHg) is independently associated with adverse outcomes, including mortality and hospital readmission [11,12]. However, the prognostic significance of echocardiographic findings specifically in the ARDS population requires further elucidation.

While echocardiography provides detailed cardiac imaging, traditional cardiac auscultation remains a fundamental component of physical examination. Heart sounds and murmurs detected through auscultation have long served as clinical markers of valvular pathology and hemodynamic abnormalities. However, auscultation in the ICU environment presents unique challenges, including ambient noise from ventilators and monitoring equipment, patient positioning limitations, and the technical difficulties of examining critically ill patients [13]. These factors have contributed to a perceived decline in the clinical utility of auscultation in modern intensive care practice. Nevertheless, the advent of electronic stethoscopy has renewed interest on cardiac auscultation by offering sound amplification, noise reduction, and the capability to record, store, and remotely transmit heart sounds for later analysis or expert consultation [14,15].

Electronic stethoscopes have gained particular relevance in the context of infectious disease outbreaks, including the COVID-19 pandemic, where devices that do not require direct ear contact facilitate patient examination while maintaining infection control precautions [15,16]. Beyond infection control, digital stethoscopy offers the unique advantage of creating standardized, archivable recordings that can be subjected to quantitative analysis and, increasingly, artificial intelligence-based interpretation. Deep learning algorithms applied to digital heart sounds have demonstrated the ability to detect murmurs associated with structural heart disease with a sensitivity of 85.6% and specificity of 84.4%, comparable to or exceeding clinician performance [17,18]. The integration of electronic auscultation with cloud-based storage platforms enables the creation of structured datasets linking heart sounds to clinical outcomes, potentially facilitating the development of machine learning algorithms for automated cardiac assessment and prognostication.

Despite the established roles of both auscultation and echocardiography in cardiac assessment, the correlation between these two modalities in critically ill patients with ARDS has not been systematically investigated. Furthermore, while individual echocardiographic parameters have been studied as prognostic markers, the combined predictive value of auscultatory and echocardiographic findings for survival and ICU resource utilization in ARDS remains unclear. Understanding these relationships could inform clinical practice by validating the continued utility of physical examination skills in the technology-intensive ICU environment, and by identifying specific cardiac parameters that warrant monitoring and intervention.

The aim of this study was to investigate the relationship between cardiac auscultation using electronic stethoscopy and echocardiographic findings in patients admitted to the ICU with ARDS requiring invasive mechanical ventilation. Additionally, we sought to evaluate the prognostic value of auscultatory and echocardiographic cardiac parameters for immediate survival, 90-day survival, and duration of ICU stay in this critically ill population.

## 2. Methods

The protocol of this study was approved by the ethics committee of “G. Papanikolaou” General Hospital of Thessaloniki, Greece (Reference Number 42/20-05-2020), before the initiation of enrolment, and a relative from each participant gave a written informed consent.

This prospective observational study enrolled patients admitted to the ICU with ARDS. All patients had ARDS secondary to coronavirus disease 2019 (COVID-19). ARDS was diagnosed according to the Berlin definition [19], which requires: (1) acute onset of respiratory symptoms within one week of a known clinical insult or new/worsening respiratory symptoms; (2) bilateral opacities on chest imaging not fully explained by effusions, lobar/lung collapse, or nodules; (3) respiratory failure not fully explained by cardiac failure or fluid overload; and (4) impaired oxygenation, defined as a ratio of the partial pressure of arterial oxygen to the fraction of inspired oxygen (PaO_2_/FiO_2_) ≤ 300 mmHg with a minimum positive end-expiratory pressure (PEEP) or continuous positive airway pressure (CPAP) of 5 cm H_2_O [19,20]. Severity was classified as mild (PaO_2_/FiO_2_ 201–300 mmHg), moderate (PaO_2_/FiO_2_ 101–200 mmHg), or severe (PaO_2_/FiO_2_ ≤ 100 mmHg). Additional inclusion criteria were age ≥18 years and the need for endotracheal intubation and invasive mechanical ventilation. The sole exclusion criterion was pregnancy. Since this was an original study, no sample size calculation was performed; instead, all eligible patients hospitalized in the 1st ICU of the “George Papanikolaou” hospital of Thessaloniki between June 2020 and June 2021 were included in the study.

An electronic stethoscope, with no need for ear contact, was used for cardiac auscultation recording and online storage in a dataset for later hearing [3]. Four recordings, lasting 15 s each, were obtained by each participant, via the positioning of the electronic stethoscope in the specific chest points of valvular (aortic, pulmonic, tricuspid, mitral) auscultation [21]. Abnormal sounds were characterized as aortic stenosis or aortic, pulmonic, tricuspid or mitral regurgitations, respectively (audio 1–5) [22]. Right after the auscultation recordings, a comprehensive transthoracic echocardiographic examination was performed at bedside by an experienced intensivist certified in critical care echocardiography, in accordance with the guidelines of the European Society of Echocardiography for performing a comprehensive transthoracic echocardiographic examination in adults [10]. All echocardiographic assessments were performed using a standard cardiac ultrasound system equipped with a phased-array transducer (2–4 MHz). Standard parasternal long-axis, parasternal short-axis, apical four-chamber, apical two-chamber, and subcostal views were obtained. Left ventricular (LV) systolic function was assessed by visual estimation of LV ejection fraction (LVEF) from multiple views, as recommended [10]. LV diastolic function was evaluated, using pulsed-wave Doppler of mitral inflow to measure early (E) and late (A) diastolic filling velocities, and tissue Doppler imaging of the medial mitral annulus to measure early diastolic annular velocity (e’). The E/e’ ratio was calculated as a surrogate of LV filling pressures. Diastolic dysfunction was graded as Grade I (impaired relaxation), Grade II (pseudonormal filling), or Grade III (restrictive filling) based on established EACVI criteria [10]. Valvular regurgitation was assessed using color Doppler imaging in multiple views and graded semi-quantitatively as none (–), mild (1+), moderate (2+), or severe (3+), based on regurgitant jet area relative to the receiving chamber area, vena contracta width, and flow convergence characteristics, in accordance with recommendations for the evaluation of native valvular regurgitation [10]. Maximal aortic valve velocity (AoVmax) was measured using continuous-wave Doppler interrogation across the aortic valve from the apical five-chamber or right parasternal view to screen for aortic stenosis. Right ventricular systolic pressure (RVSP) was estimated from the peak tricuspid regurgitation (TR) jet velocity using continuous-wave Doppler, applying the simplified Bernoulli equation (RVSP = 4V^2^ + estimated right atrial pressure [RAP]), where V represents the peak TR jet velocity [10]. RAP was estimated from the inferior vena cava (IVC) diameter and its respiratory variation (collapsibility index), as recommended by the guidelines: an IVC diameter ≤2.1 cm that collapsed >50% with a sniff corresponded to a normal RAP of 3 mmHg (range 0–5 mmHg); an IVC diameter >2.1 cm with <50% collapse corresponded to an elevated RAP of 15 mmHg (range 10–20 mmHg); intermediate scenarios were assigned an RAP of 8 mmHg (range 5–10 mmHg) [10]. In cases where the TR jet was absent or the Doppler signal was insufficient to obtain a reliable peak velocity, RVSP could not be estimated non-invasively and was recorded as missing for that patient. This is a recognized limitation of the non-invasive estimation of pulmonary artery pressures, as the absence of a measurable TR jet does not exclude pulmonary hypertension [10]. The IVC was assessed from the subcostal view, and its maximum diameter was measured in the longitudinal plane, approximately 1–2 cm from the junction with the right atrium, at end-expiration. The presence or absence of pericardial effusion was also documented [23].

Apart from that, participants’ baseline characteristics such as age, gender, Charlson comorbidity index [24], acute physiology and chronic health evaluation II (APACHE II) score [25], and daily sequential organ failure assessment (SOFA) score [26], as well as their duration of ICU stay and their survival and 90-day survival, were also recorded.

Heart sounds were interpreted separately by two consultants of Cardiology, who were blinded to the topic of the study and to the ultrasonographic findings, and who had at least 20 years of clinical experience in a Cardiology Department after obtaining the title of Cardiologist and were working outside of the ICU at the time of the study. Cases of disagreement between those two were solved by a third consultant with the same profile.

Recordings were performed at admission and repeated each time the treating physician identified a significant change in the patient’s clinical status. Each auscultation recording session was immediately followed by echocardiography. Both the auscultation recording and the echocardiographic examination were performed by the same experienced intensivist. Two intensivists with this profile participated in the study.

Statistical analysis was performed using the SPSS (version-20 IBM-SPSS-statistical-software, Armonk, NY, USA). Continuous variables are presented as mean ± standard deviation (SD) and categorical variables as number/total (%). Shapiro–Wilk test was used to investigate for normality. To assess differences in echocardiographic variables between patients with and without auscultatory abnormalities on cardiac auscultation, an independent-samples t-test and Mann–Whitney U test were used for parametric and non-parametric variables, respectively. To search for predictors of survival and 90-day survival, univariate logistic regression analysis was used, followed by a multivariate logistic regression analysis, using the backward likelihood ratio, among the variables that were significant in the univariate one. Finally, to investigate for variables that affected the duration of ICU stay, univariate linear regression analysis was used, followed by multivariate linear regression analysis among the factors that were significant in the univariate analysis. All tests were two-sided and *p* < 0.05 was accepted as statistically significant.

## 3. Results

Table 1 shows the baseline characteristics of the cohort. The relationship between cardiac auscultation and echocardiography is shown in Table 2. Maximal aortic valve velocity (AoVmax) was significantly higher in patients with an aortic stenosis murmur on cardiac auscultation than in those without (1.79 ± 0.80 m/s vs. 1.05 ± 0.08 m/s, *p* = 0.009). Similarly, echocardiographic mitral and tricuspid regurgitation severity was significantly greater in patients with corresponding regurgitation murmurs on cardiac auscultation (mitral: 1.69 ± 0.75 vs. 0.24 ± 0.43 grade, *p* < 0.001; tricuspid: 2.14 ± 0.52 vs. 0.30 ± 0.46 grade, *p* < 0.001). Patients with a mitral regurgitation murmur also had significantly higher RVSP (48.08 ± 7.87 vs. 39.64 ± 12.15 mmHg, *p* = 0.023) and wider IVC diameter (2.29 ± 0.38 vs. 1.91 ± 0.41 cm, *p* = 0.002). Likewise, patients with a tricuspid regurgitation murmur had higher RVSP (50.94 ± 9.54 vs. 35.66 ± 9.55 mmHg, *p* < 0.001) and wider IVC diameter (2.16 ± 0.39 vs. 1.90 ± 0.42 cm, *p* = 0.003).

In univariate logistic regression analysis (Table 3), increasing age (odds ratio [OR] 1.09, 95% confidence interval [CI] 1.05–1.13, *p* < 0.001), Charlson comorbidity index (OR 1.54, 95% CI 1.26–1.88, *p* < 0.001), APACHE II score (OR 1.13, 95% CI 1.07–1.20, *p* < 0.001), mean daily SOFA score (OR 2.09, 95% CI 1.64–2.68, *p* < 0.001), and RVSP (OR 1.05, 95% CI 1.01–1.09, *p* = 0.007) were significantly associated with increased ICU mortality. Increasing severity of aortic regurgitation on echocardiography also increased the odds of death (OR 3.15, 95% CI 1.20–8.27, *p* = 0.020), while higher ejection fraction was protective (OR 0.93, 95% CI 0.88–0.98, *p* = 0.006).

For 90-day mortality (Table 4), significant univariate predictors included increasing age (OR 1.10, 95% CI 1.05–1.14, *p* < 0.001), Charlson comorbidity index (OR 1.84, 95% CI 1.45–2.34, *p* < 0.001), APACHE II score (OR 1.18, 95% CI 1.10–1.27, *p* < 0.001), mean daily SOFA score (OR 1.94, 95% CI 1.54–2.43, *p* < 0.001), AoVmax (OR 16.63, 95% CI 1.03–269.72, *p* = 0.048), and RVSP (OR 1.06, 95% CI 1.02–1.10, *p* = 0.002). The presence of a mitral regurgitation murmur on cardiac auscultation (OR 4.29, 95% CI 1.13–16.31, *p* = 0.033) and increasing echocardiographic severity of mitral regurgitation (OR 2.09, 95% CI 1.21–3.62, *p* = 0.008) and tricuspid regurgitation (OR 1.49, 95% CI 1.03–2.14, *p* = 0.033) also increased the risk. Higher ejection fraction remained protective (OR 0.93, 95% CI 0.88–0.98, *p* = 0.006).

In multivariate logistic regression analysis (Table 5), mean daily SOFA score (OR 2.39, 95% CI 1.57–3.64, *p* < 0.001) and RVSP (OR 1.07, 95% CI 1.02–1.11, *p* = 0.006) were the only independent predictors of ICU mortality. For 90-day mortality, independent predictors were APACHE II score (OR 1.25, 95% CI 1.07–1.47, *p* = 0.006), mean daily SOFA score (OR 1.54, 95% CI 1.02–2.31, *p* = 0.039), RVSP (OR 1.07, 95% CI 1.01–1.13, *p* = 0.020), and echocardiographic mitral regurgitation severity (OR 2.98, 95% CI 1.11–8.01, *p* = 0.031).

Regarding ICU length of stay (Table 6), female gender was associated with a shorter duration (r = −0.20, *p* = 0.042) in the univariate analysis, while an increasing mean daily SOFA score (r = 0.38, *p* < 0.001), tricuspid regurgitation murmur on cardiac auscultation (r = 0.26, *p* = 0.013), and increasing echocardiographic tricuspid regurgitation severity (r = 0.23, *p* = 0.024) predicted a longer ICU stay. In multivariate linear regression analysis, only the mean daily SOFA score (r = 0.35, *p* < 0.001) and echocardiographic tricuspid regurgitation severity (r = 0.25, *p* = 0.012) remained significant independent predictors.

## 4. Discussion

The present study investigated the relationship between cardiac auscultation and echocardiography in critically ill patients with ARDS requiring mechanical ventilation and examined their prognostic value for survival and ICU length of stay. Our findings demonstrate a significant correlation between auscultatory abnormalities and corresponding echocardiographic parameters. Most importantly, we identified that right ventricular systolic pressure (RVSP) and mean daily Sequential Organ Failure Assessment (SOFA) score were independent predictors of both immediate and 90-day mortality, while mitral regurgitation detected on echocardiography provided additional prognostic information for long-term survival.

A key finding of our study was the significant correlation between cardiac auscultation and echocardiographic findings. Patients with auscultatory evidence of aortic stenosis demonstrated significantly higher maximal aortic valve velocities on echocardiography, confirming the validity of bedside cardiac examination even in the challenging ICU environment. Similarly, mitral and tricuspid regurgitation murmurs correlated strongly with the degree of regurgitation visualized on color Doppler echocardiography. Furthermore, the presence of atrioventricular valve regurgitation on auscultation was associated with elevated RVSP and dilated inferior vena cava, suggesting that auscultatory findings can serve as a surrogate marker for right heart strain and pulmonary hypertension. These observations support the continued utility of cardiac auscultation as a bedside screening tool in the ICU, particularly in settings where immediate echocardiographic assessment may not be available [23,27].

The use of an electronic stethoscope with online storage capability in our study represents an innovative approach to cardiac monitoring in critically ill patients. Digital stethoscopy offers several advantages in the ICU setting, including sound amplification, noise reduction or cancelation, and the ability to record and review auscultatory findings [14,28]. This technology has gained particular relevance during the COVID-19 pandemic, where ear-contactless devices facilitate examination while maintaining infection control measures [16]. Crucially, the digital nature of these recordings creates structured datasets that are amenable to artificial intelligence (AI) analysis, opening new frontiers in automated cardiac assessment and prognostication [29].

The integration of AI and machine learning algorithms with electronic auscultation represents a paradigm shift in cardiac assessment. Our study generated a dataset of digitally recorded heart sounds with corresponding echocardiographic validation and clinical outcomes, which constitutes an ideal substrate for developing AI-driven diagnostic tools. Machine learning models can be trained to automatically detect and classify cardiac murmurs, identify valvular abnormalities, and potentially predict adverse outcomes based on acoustic features imperceptible to the human ear [30]. Recent advances in deep learning, particularly convolutional neural networks (CNNs) and recurrent neural networks (RNNs), have demonstrated remarkable accuracy in heart sound classification. Notably, FDA-cleared algorithms have achieved sensitivity of 85.6% and specificity of 84.4% for detecting structural murmurs, with performance improving to 97.9% sensitivity for clearly audible murmurs in adults—substantially outperforming average clinician accuracy of 77.9% [18]. The correlation we observed between auscultatory findings and echocardiographic parameters provides the ground truth necessary for supervised learning approaches.

Beyond diagnostic classification, AI algorithms could leverage the acoustic signatures captured in our recordings to develop novel prognostic models. The frequency spectrum analysis of heart sounds, including spectral centroid, dominant frequencies, and energy distribution across frequency bands, may contain prognostic information that complements traditional severity scores [31,32]. Our finding that RVSP independently predicts mortality suggests that acoustic markers of right heart strain and pulmonary hypertension could be detected through AI analysis of heart sounds. An AI system trained on phonocardiographic data linked to outcomes could potentially identify patients at high risk of mortality or prolonged ICU stay in real-time, enabling earlier intervention and more personalized care. Such a system would be particularly valuable in resource-limited settings where echocardiography may not be readily available [17,33].

The identification of RVSP as an independent predictor of mortality in ARDS patients is consistent with the growing body of evidence highlighting the importance of right ventricular function in critically ill patients [34,35]. Pulmonary hypertension and right ventricular dysfunction are common complications of ARDS, resulting from hypoxic pulmonary vasoconstriction, lung parenchymal destruction, and the effects of positive pressure ventilation [36]. A systematic review and meta-analysis by Sato et al. demonstrated that right ventricular injury in ARDS is significantly associated with increased mortality (OR = 1.68, 95% CI = 1.21–2.32) [5]. The prevalence of acute cor pulmonale in moderate-to-severe ARDS ranges from 20% to 50%, with echocardiographically evident right ventricular dysfunction being one of the major determinants of mortality [37,38]. Our findings extend these observations, with RVSP remaining a significant predictor of death even after adjusting for disease severity scores and other echocardiographic parameters. The mean RVSP in our cohort was 40.81 ± 12.00 mmHg, and each unit increase in RVSP was associated with a 7% increased risk of mortality and 90-day mortality. This suggests that monitoring pulmonary pressures and implementing strategies to reduce right ventricular afterload may be beneficial in ARDS management [39].

As expected, the mean daily SOFA score emerged as a robust predictor of outcomes in our cohort. The SOFA score is a validated tool for assessing organ dysfunction and predicting mortality in ICU patients [26]. A recent systematic review and meta-analysis evaluating prediction models for mortality in moderate-to-severe ARDS demonstrated that the SOFA score has good discriminatory ability, with a pooled area under the curve (AUC) of 0.802 (95% CI = 0.719–0.885) [40]. In our multivariate analysis, each unit increase in mean daily SOFA score was associated with more than a two-fold increase in the odds of mortality. The combination of the SOFA score with cardiac ultrasound parameters, particularly RVSP and mitral regurgitation severity, may provide enhanced prognostic stratification compared to severity scores alone. AI algorithms could potentially integrate these multiple data streams—auscultatory recordings, echocardiographic parameters, and clinical severity scores—into unified predictive models with superior discriminatory performance [41].

An interesting finding of our study was the independent association between mitral regurgitation severity on echocardiography and 90-day mortality. While mitral regurgitation on auscultation was significant in univariate analysis, only the ultrasound-detected regurgitation retained significance in the multivariate model. This observation suggests that the degree of regurgitation quantified by color Doppler provides more precise prognostic information than auscultatory detection alone. However, AI-enhanced auscultation could potentially bridge this gap by extracting quantitative features from heart sound recordings that correlate with regurgitation severity, effectively transforming a qualitative bedside assessment into a semi-quantitative screening tool [42]. Mitral regurgitation in ARDS may reflect elevated left atrial pressures, left ventricular dysfunction, or structural changes secondary to critical illness cardiomyopathy. The association with 90-day mortality rather than immediate survival suggests that mitral regurgitation may be a marker of longer-term cardiac dysfunction that affects recovery and rehabilitation outcomes.

Regarding ICU length of stay, our analysis revealed that the mean daily SOFA score and tricuspid regurgitation severity on echocardiography were independent predictors of a prolonged ICU admission among survivors. The association between tricuspid regurgitation and extended ICU stay likely reflects the impact of right ventricular dysfunction on hemodynamic stability and weaning from mechanical ventilation [36]. Right heart failure can impede fluid management, limit positive end-expiratory pressure optimization, and complicate the transition to spontaneous breathing [43]. The finding that female gender was associated with shorter ICU stays in univariate analysis, though not independently significant, may warrant further investigation in larger cohorts.

The digital infrastructure established in our study—electronic stethoscopy with cloud-based storage—creates opportunities for AI-enabled continuous cardiac monitoring. Unlike traditional auscultation, which provides only intermittent snapshots of cardiac status, wearable or bed-mounted digital stethoscopes could provide continuous phonocardiographic monitoring with real-time AI analysis [44,45]. Such systems could detect the onset of new murmurs, track changes in heart sound intensity or timing, and alert clinicians to deteriorating cardiac function before it becomes clinically apparent. This concept of intelligent acoustic monitoring aligns with the broader movement toward precision medicine and early warning systems in critical care [46]. The CoCross platform utilized in our study for recording and storage provides a foundation upon which such AI-enhanced monitoring systems could be developed [21].

The integration of lung ultrasound and echocardiography has become increasingly recognized as an essential component of critical care assessment. International guidelines now recommend point-of-care ultrasound as a standard tool for ICU management, and recent consensus statements have outlined the basic skills required for intensivists [47,48]. Our study adds to this evidence base by demonstrating that echocardiographic parameters, particularly those reflecting right heart function and pulmonary hemodynamics, provide prognostic information beyond traditional severity scores. The combination of auscultation and ultrasound creates a comprehensive bedside assessment that can guide clinical decision-making regarding fluid management, vasopressor therapy, and ventilator settings [49].

The clinical implications of our findings are multifold. First, the correlation between auscultation and echocardiography validates the continued use of physical examination skills in the technologically advanced ICU environment. Second, the identification of RVSP as an independent mortality predictor emphasizes the importance of routine echocardiographic assessment in ARDS patients, with particular attention to right ventricular function and pulmonary pressures. Third, serial monitoring of cardiac function may help identify patients at risk of adverse outcomes who might benefit from intensified therapy or consideration for advanced support. Fourth, the use of electronic stethoscopy offers opportunities for standardized cardiac monitoring, remote consultation, and quality improvement initiatives in critical care [50]. Finally, and perhaps most significantly, the dataset generated by our study provides a foundation for developing AI-powered clinical decision support systems that could democratize expert-level cardiac assessment across diverse healthcare settings [51].

The convergence of electronic auscultation and AI has profound implications for telemedicine and healthcare delivery in underserved areas [52,53]. An AI algorithm capable of detecting valvular abnormalities and predicting outcomes from heart sound recordings could be deployed on smartphones or portable devices, enabling frontline healthcare workers to obtain specialist-level cardiac assessments without the need for expensive echocardiography equipment or on-site cardiologist expertise [54]. Recently, the DAMSUN-HF study demonstrated that AI-enabled digital stethoscopes could detect heart failure with reduced ejection fraction with 96.9% sensitivity in resource-limited settings in Ghana, illustrating the transformative potential of this technology [55]. In the context of ARDS, such technology could facilitate triage decisions, guide transfers to tertiary centers, and support remote intensivist consultations. Our study demonstrates that auscultatory findings correlate with clinically meaningful echocardiographic parameters and outcomes, establishing the clinical validity that would be essential for regulatory approval and clinical adoption of AI-based auscultation tools [56].

Several limitations of our study should be acknowledged. First, this was a single-center study conducted in one ICU, which may limit the generalizability of our findings to other settings and populations. Second, the sample size, while adequate for detecting significant associations, may have been insufficient to identify smaller effect sizes or to perform more complex subgroup analyses, and would be considered modest for training robust machine learning models. Third, cardiac assessments were performed at a single time point upon ICU admission, and serial measurements might have provided additional prognostic information regarding trends and response to treatment. Fourth, the study was conducted during a period that included the COVID-19 pandemic, and although ARDS patients of various etiologies were included, the predominance of viral pneumonia cases may have influenced the results. Fifth, inter-observer variability in both auscultation interpretation and echocardiographic measurements was not formally assessed, although examinations were performed by experienced practitioners. Finally, while our study establishes the feasibility and clinical correlations of digital auscultation, we did not directly implement or validate AI algorithms on the collected recordings, which represents an important next step.

Future research should prioritize the development and validation of AI algorithms using the phonocardiographic data generated by studies such as ours. The integration of auscultatory AI with other data streams—including vital signs, laboratory values, ventilator parameters, and imaging—could yield multimodal predictive models with unprecedented accuracy [57]. Prospective studies should evaluate whether AI-assisted auscultation improves clinical decision-making, patient outcomes, and resource utilization compared to standard care. Regulatory pathways for AI-based cardiac diagnostic tools are evolving, and studies demonstrating clinical validity and utility, such as ours, will be essential for bringing these technologies to the bedside [58].

## 5. Conclusions

This study demonstrates that cardiac auscultation using electronic stethoscopy correlates significantly with echocardiographic findings in mechanically ventilated ARDS patients. Right ventricular systolic pressure and the mean daily SOFA score are independent predictors of mortality, while mitral regurgitation severity on echocardiography provides additional prognostic information for 90-day survival. Tricuspid regurgitation severity predicts a prolonged ICU length of stay among survivors.

## Figures and Tables

**Table 1:** Baseline characteristics of the cohort.

Characteristic			Value
Age (years)			65.41 ± 10.16
Gender	Female		57/173 (32.9%)
	Male		116/173 (67.1%)
Charlson comorbidity index			3.26 ± 1.98
APACHE II score			16.40 ± 6.39
Mean daily SOFA score			5.76 ± 2.16
Cardiac auscultation	Aortic stenosis		12/173 (6.9%)
	Aortic regurgitation		1/173 (0.6%)
	Pulmonic regurgitation		3/173 (1.7%)
	Mitral regurgitation		13/173 (7.5%)
	Tricuspid regurgitation		29/173 (16.8%)
Echocardiography	Ejection fraction (%)		52.68 ± 6.68
	E/E’		10.92 ± 3.09
	Diastolic dysfunction	None	6/88 (6.8%)
		Grade I	55/88 (62.5%)
		Grade II	22/88 (25.0%)
		Grade III	5/88 (5.7%)
	AoVmax (m/s)		1.46 ± 0.63
	Aortic regurgitation	-	152/173 (87.9%)
		1+	21/173 (12.1%)
		2+	0/173 (0.0%)
		3+	0/173 (0.0%)
	Mitral regurgitation	-	126/173 (72.8%)
		1+	37/173 (21.4%)
		2+	9/173 (5.2%)
		3+	1/173 (0.6%)
	Tricuspid regurgitation	-	98/173 (56.6%)
		1+	45/173 (26.0%)
		2+	24/173 (13.9%)
		3+	6/173 (3.5%)
	RVSP (mmHg)		40.81 ± 12.00
	IVC (cm)		1.95 ± 0.41
	Pericardial effusion		29/173 (16.8%)
Survival	Yes		100/173 (57.8%)
	No		73/173 (42.2%)
Days in ICU			21.09 ± 12.59 (N = 100)
90-day survival	Yes		92/173 (53.2%)
	No		81/173 (46.8%)

APACHE II = acute physiology and chronic health evaluation II; SOFA: sequential organ failure assessment; AoVmax = maximal aortic valve velocity; m = meters; s = seconds; RVSP = right ventricular systolic pressure; mmHg = millimeters of Mercury; IVC = inferior vena cava; cm = centimeters; ICU = intensive care unit.

**Table 2:** Relationship between cardiac auscultation and echocardiography.

Aortic stenosis			
Echocardiographic variable	No	Yes	*p* (value)
Ejection fraction (%)	52.89 ± 6.95	51.96 ± 5.67	0.65
E/E’	10.95 ± 3.17	10.12 ± 3.27	0.55
Diastolic dysfunction (grade)	1.34 ± 0.68	1.56 ± 1.01	0.55
AoVmax (m/s)	1.05 ± 0.08	1.79 ± 0.80	0.009
RVSP (mmHg)	40.22 ± 12.18	39.50 ± 6.46	0.86
IVC (cm)	1.96 ± 0.43	1.90 ± 0.35	0.69
Mitral regurgitation			
Echocardiographic variable	No	Yes	*p* (value)
Ejection fraction (%)	53.30 ± 6.51	47.09 ± 8.68	0.002
E/E’	10.83 ± 3.08	11.72 ± 4.05	0.47
Diastolic dysfunction (grade)	1.37 ± 0.71	1.36 ± 0.81	0.99
Mitral regurgitation severity (echocardiographic grade)	0.24 ± 0.43	1.69 ± 0.75	<0.001
RVSP (mmHg)	39.64 ± 12.15	48.08 ± 7.87	0.023
IVC (cm)	1.91 ± 0.41	2.29 ± 0.38	0.002
Tricuspid regurgitation			
Echocardiographic variable	No	Yes	*p* (value)
Ejection fraction (%)	53.23 ± 7.08	50.45 ± 5.34	0.05
E/E’	11.30 ± 2.55	10.39 ± 4.56	0.52
Diastolic dysfunction (grade)	1.32 ± 0.68	1.37 ± 0.83	0.78
Tricuspid regurgitation severity (echocardiographic grade)	0.30 ± 0.46	2.14 ± 0.52	<0.001
RVSP (mmHg)	35.66 ± 9.55	50.94 ± 9.54	<0.001
IVC (cm)	1.90 ± 0.42	2.16 ± 0.39	0.003

AoVmax = maximal aortic valve velocity; m = meters; s = seconds; RVSP = right ventricular systolic pressure; mmHg = millimeters of Mercury; IVC = inferior vena cava; cm = centimeters.

**Table 3:** Factors that affected survival.

Variable	OR (95% C.I.)	*p* (Value)
Age (years)	1.09 (1.05–1.13)	<0.001
Gender (female)	0.72 (0.38–1.38)	0.32
Charlson comorbidity index	1.54 (1.26–1.88)	<0.001
APACHE II score	1.13 (1.07–1.20)	<0.001
Mean daily SOFA score	2.09 (1.64–2.68)	<0.001
Aortic stenosis on cardiac auscultation	0.47 (0.12–1.84)	0.28
Mitral regurgitation on cardiac auscultation	1.25 (0.40–3.94)	0.7
Tricuspid regurgitation on cardiac auscultation	1.24 (0.55–2.79)	0.61
Ejection fraction (%)	0.93 (0.88–0.98)	0.006
E/E’	0.99 (0.83–1.19)	0.93
Diastolic dysfunction (grade)	0.59 (0.30–1.15)	0.12
AoVmax (m/s)	2.90 (0.70–11.94)	0.14
Aortic regurgitation severity (echocardiographic grade)	3.15 (1.20–8.27)	0.02
Mitral regurgitation severity (echocardiographic grade)	1.26 (0.76–2.07)	0.37
Tricuspid regurgitation severity (echocardiographic grade)	1.19 (0.83–1.69)	0.35
RVSP (mmHg)	1.05 (1.01–1.09)	0.007
IVC (cm)	0.58 (0.26–1.30)	0.18
Pericardial effusion	0.81 (0.36–1.83)	0.61

OR = odds ratio; C.I. = confidence intervals; APACHE II = acute physiology and chronic health evaluation II; SOFA: sequential organ failure assessment; AoVmax = maximal aortic valve velocity; m = meters; s = seconds; RVSP = right ventricular systolic pressure; mmHg = millimeters of Mercury; IVC = inferior vena cava; cm = centimeters.

**Table 4:** Factors that affected 90-day survival.

Variable	OR (95% C.I.)	*p* (Value)
Age (years)	1.10 (1.05–1.14)	<0.001
Gender (female)	0.75 (0.40–1.43)	0.38
Charlson comorbidity index	1.84 (1.45–2.34)	<0.001
APACHE II score	1.18 (1.10–1.27)	<0.001
Mean daily SOFA score	1.94 (1.54–2.43)	<0.001
Aortic stenosis on cardiac auscultation	0.84 (0.25–2.78)	0.77
Mitral regurgitation on cardiac auscultation	4.29 (1.13–16.31)	0.033
Tricuspid regurgitation on cardiac auscultation	2.25 (0.98–5.17)	0.06
Ejection fraction (%)	0.93 (0.88–0.98)	0.006
E/E’	1.01 (0.84–1.20)	0.94
Diastolic dysfunction (grade)	0.81 (0.43–1.51)	0.51
AoVmax (m/s)	16.63 (1.03–269.72)	0.048
Aortic regurgitation severity (echocardiographic grade)	2.54 (0.97–6.64)	0.06
Mitral regurgitation severity (echocardiographic grade)	2.09 (1.21–3.62)	0.008
Tricuspid regurgitation severity (echocardiographic grade)	1.49 (1.03–2.14)	0.033
RVSP (mmHg)	1.06 (1.02–1.10)	0.002
IVC (cm)	0.67 (0.31–1.49)	0.33
Pericardial effusion	0.77 (0.34–1.72)	0.52

APACHE II = acute physiology and chronic health evaluation II; SOFA: sequential organ failure assessment; AoVmax = maximal aortic valve velocity; m = meters; s = seconds; RVSP = right ventricular systolic pressure; mmHg = millimeters of Mercury; IVC = inferior vena cava; cm = centimeters.

**Table 5:** Multivariate logistic regression analysis for survival and 90-day survival.

Survival		
Variable	OR (95% C.I.)	*p* (value)
Age (years)	1.03 (0.96–1.10)	0.41
Charlson comorbidity index	0.92 (0.64–1.31)	0.63
APACHE II score	1.08 (0.98–1.19)	0.11
Mean daily SOFA score	2.39 (1.57–3.64)	<0.001
Ejection fraction (%)	0.96 (0.87–1.06)	0.47
Aortic regurgitation severity (echocardiographic grade)	1.33 (0.28–6.18)	0.72
RVSP (mmHg)	1.07 (1.02–1.11)	0.006
90-day survival		
Variable	OR (95% C.I.)	*p* (value)
Age (years)	1.01 (0.91–1.12)	0.88
Charlson comorbidity index	1.34 (0.83–2.17)	0.23
APACHE II score	1.25 (1.07–1.47)	0.006
Mean daily SOFA score	1.54 (1.02–2.31)	0.039
Mitral regurgitation on cardiac auscultation	0.59 (0.04–9.34)	0.71
Ejection fraction (%)	1.02 (0.90–1.16)	0.75
AoVmax (m/s)	n/a	n/a
Mitral regurgitation severity (echocardiographic grade)	2.98 (1.11–8.01)	0.031
Tricuspid regurgitation severity (echocardiographic grade)	0.47 (0.16–1.35)	0.16
RVSP (mmHg)	1.07 (1.01–1.13)	0.02

OR = odds ratio; C.I. = confidence intervals; APACHE II = acute physiology and chronic health evaluation II; SOFA: sequential organ failure assessment; RVSP = right ventricular systolic pressure; mmHg = millimeters of Mercury; AoVmax = maximal aortic valve velocity; m = meters; s = seconds; n/a = not applicable.

**Table 6:** Univariate and multivariate linear regression analyses among factors that correlated with the days of ICU stay.

Univariate analysis			
Variable	B (95% C.I.)	r	*p* (value)
Age (years)	0.16 (−0.09–0.41)	0.13	0.21
Gender (female)	−5.31 (−10.43–−0.18)	−0.20	0.042
Charlson comorbidity index	0.41 (−1.07–1.89)	0.06	0.59
APACHE II score	0.35 (−0.12–0.81)	0.15	0.14
Mean daily SOFA score	2.91 (1.48–4.33)	0.38	<0.001
Aortic stenosis on cardiac auscultation	−5.85 (−13.52–1.82)	−0.17	0.13
Mitral regurgitation on cardiac auscultation	4.32 (−4.45–13.09)	0.11	0.33
Tricuspid regurgitation on cardiac auscultation	8.41 (1.78–15.03)	0.26	0.013
Ejection fraction (%)	−0.15 (−0.61–0.31)	−0.07	0.53
E/E’	−0.10 (−1.49–1.30)	−0.03	0.89
Diastolic dysfunction (grade)	−0.96 (−5.99–4.08)	−0.05	0.71
AoVmax (m/s)	−1.15 (−21.73–19.42)	−0.03	0.91
Aortic regurgitation severity (echocardiographic grade)	0.36 (−9.48–10.21)	0.01	0.94
Mitral regurgitation severity (echocardiographic grade)	0.58 (−3.66–4.82)	0.03	0.79
Tricuspid regurgitation severity (echocardiographic grade)	3.41 (0.45–6.38)	0.23	0.024
RVSP (mmHg)	0.37 (−0.02–0.75)	0.24	0.06
IVC (cm)	2.97 (−4.12–10.06)	0.09	0.41
Pericardial effusion	2.06 (−4.47–8.58)	0.06	0.53
Multivariate analysis			
Variable	B (95% C.I.)	r	*p* (value)
Gender (female)	−3.85 (−8.92–1.23)	−0.15	0.14
Mean daily SOFA score	2.70 (1.23–4.18)	0.35	<0.001
Tricuspid regurgitation on cardiac auscultation	4.13 (−6.89–15.14)	0.13	0.46
Tricuspid regurgitation severity (echocardiographic grade)	3.73 (0.85–6.61)	0.25	0.012

C.I. = confidence intervals; APACHE II = acute physiology and chronic health evaluation II; SOFA: sequential organ failure assessment; AoVmax = maximal aortic valve velocity; m = meters; s = seconds; RVSP = right ventricular systolic pressure; mmHg = millimeters of Mercury; IVC = inferior vena cava; cm = centimeters.

## Data Availability

The data presented in this study are available on request from the corresponding author due to institutional restrictions.
